# Supplementary material for: Primary postpartum haemorrhage and longer-term physical, psychological, and psychosocial health outcomes for women and their partners in high income countries: A mixed-methods systematic review
Source: PLoS One. 2023 Jun 14;18(6):e0274041. doi: 10.1371/journal.pone.0274041 (PMC10266652; doi:10.1371/journal.pone.0274041)
Supplement: Attachment — Submitted filename: Response to reviewers_PLOSONE_7 Dec 22.docx [file pone.0274041.s005.docx]

**Response to reviewers’ comment**

| **Editor/reviewer comment** | **Response** |
| --- | --- |
| **Reviewer #1** |  |
| This systematic review of quantitative and qualitative studies on the long-term outcomes of postpartum haemorrhage is well written. | Thank you. |
| Two limitations of the review are: firstly, it includes only publications in English and secondly it includes only publications from high income countries. Globally postpartum haemorrhage is a more significant cause of maternal mortality in low- and middle-income countries which contribute to a greater share of the global burden of maternal morbidity among survivors. | We recognise the importance of PPH in low- and middle-income countries. This review was carried out as part of a bigger project exploring longer-term outcomes of primary PPH in a UK population, with the rationale that the incidence of PPH is increasing in the UK and in other high income countries. We have given more information about the rationale in the introduction and added acknowledgement of this limitation and an explanation to the Strengths and Limitations section. |
| The authors should explain the rationale for excluding publications from low- and middle-income countries in their review. I did not find country status as a term in the search strategy file (S1 File). | We have added more information under Strengths and Limitations and strengthened the rationale for focusing on high-income countries in the Introduction. Restriction to high income countries was done by including high-income countries as an eligibility criterion during study selection. Search terms were not restricted based on countries to minimise excluding relevant articles. We have included this explanation in the Methods section. |
| Furthermore, the authors should consider revising the title to “Primary postpartum haemorrhage and longer-term physical, psychological, and psychosocial health outcomes for women and their partners in high income countries: a mixed-methods systematic review”. | Thank you for this suggestion. We have revised the title. |
| **Reviewer #2** |  |
| Post-partum hemorrhage is one of the main complications of childbirth worldwide and most research have been focusing on its immediate outcomes on women. This original research aims at summarizing the literature on long-term outcomes of post-partum hemorrhage (PPH) in high-income countries, on women and their partners; an objective we consider to be important for both clinical settings and policy implications. The main objective is coherent, the methods (mix-methods systematic review) is relevant, and the expected results can be significant for maternal health in high income countries. Considering women’s experience of PPH and the long-term outcomes of the partner are also strong aspects of this review | Thank you, we appreciate the reviewer’s positive comments about the study. |
| In the article, the authors have properly placed the claims in the context of the previous literature and clearly explained why this review is needed in view of the current knowledge on the subject. Yet, we think the rationale could be emphasized and could further describe why it is important to obtain data on long-term outcomes following a post-partum hemorrhage (PPH). Indeed, the problem is well defined but does not describe the importance of getting such results. | Thank you for pointing out this gap in the introduction. We have revised the paper with this in mind. (l61-67 of unmarked manuscript) |
| Besides, we think that exploring women’s experience of PPH is crucial and is a strength of this article but the part on experiences of care does not directly answer to the main objective of this review. Indeed, we consider that this part of the study (especially the experiences of care – see inclusion criteria for qualitative studies / outcome) answers to a different and specific objective than synthesizing the evidence about the long-term outcomes of PPH, and we recommend a clarification on this matter. | It was always a secondary objective of this review, set out in the PROSPERO protocol, to explore and include women’s experiences of care, with the rationale that this might help understand some longer-term health outcomes, particularly in relation to mental health. This secondary objective, and the rationale for this, was not clearly articulated in the original draft of the paper. We have added to the introduction to provide a clearer rationale as well as making the secondary objective clear. |
| Moreover, the systematic review focuses on longer-term outcomes of PPH with the main objective being to “synthesize the evidence about the long-term (…) consequences of PPH”. Yet, the inclusion criteria presented in table 1 consider that studies could be included with any follow-up duration after hospital discharge following PPH. Could we consider outcomes occurring at hospital discharge to be long-term outcomes (see table 4 \| study 33 – Drayton el al. 2016 and study 32 – Chessman et al 2018)? | The inclusion of studies investigating outcomes occurring shortly after hospital discharge was set out a priori in our protocol. The rationale for this was two-fold. First, there is no recognised definition of what is considered to be a ‘longer-term’ outcome. Second, previous reviews have provided some evidence of post-discharge outcomes that might have an impact on longer-term wellbeing. Therefore, in order to maximise inclusion, and avoid an arbitrary cut-off for ‘longer-term’, we chose to include any post-discharge outcome. |
| The protocol of the review is available on Prospero. Some minor changes from the initial protocol have been observed. Indeed, the protocol mentioned that “There will be no limitations on language” but the final manuscript states as exclusion criteria “Papers which were not written in English”. | Thank you for checking the PROSPERO protocol. We would like to clarify that language restrictions were not made in the search strategy. English language was included as an eligibility criterion during the study selection stage, based on the advice of the senior outreach librarian at the University of Oxford Health Care Libraries (and see <https://www.jclinepi.com/article/S0895-4356(20)31238-5/fulltext>). This approach was agreed a priori but was unintentionally not included in the PROSPERO protocol. We have clarified the approach used to exclude non-English language papers in the Methods section. |
| Moreover, the protocol mentioned “The search for unpublished studies and grey literature will include reports from WHO, UNFPA, NHS Maternity statistics. » but the article mentions no grey literature search nor reports from WHO, UNFPA, NHS. These changes have not been explained in the final article. Only a sentence in the conclusion explains that five articles written in another language from English have been excluded due to lack of sufficient funding. | It was our original intention to search for unpublished studies and grey literature, but due to time and funding constraints we were unable to do so. We have now referred to this variation from the protocol under Strengths and limitations. |
| Yet, these changes are minors, and we did not observe any major deviation from the initial protocol published. The mix-method of this systematic review brings substantial value to the overall results. Some choices in the search strategy are raising questions (see below) but don’t seem to compromise the validity of the results nor induce any publication bias. The risk of bias and quality assessment is coherent and well conducted and exposed. The results and discussion parts are very clear, well-structured, and organized. | Thank you for these encouraging comments and recognising that these were minor practical changes to protocol. |
| Finally, the manuscript is well organized and mostly clearly written. The introduction could benefit from revision in the writing, to clarify the link between ideas. | We have revised the introduction session to improve the structure and make clearer links between ideas. |
| The inclusion / exclusion criteria are clearly presented in a table (table 1) although it could be interesting to clarify some minor aspects (see below). The study is mostly conformed to PRISMA guidelines, except from minor details presented below. The tables are well presented and help the reader follow the different steps of the review: they are very clear. No plagiarism detected. No conflict of interest stated nor detected. | We appreciate these positive comments and constructive feedback. |
| As is, we advise the editor to accept the article with major revision. We strongly encourage the authors to revise the few changes proposed and re-submit a revised version of their article as we consider this is solid work that just needs to be consolidated to gain clarity. | Thank you. We have made a number of changes to the manuscript based on reviewers’ comments. |

**Response and changes made to proposed changes**

| **Changes suggested/Questions** | **Changes made/clarifications** |
| --- | --- |
| ***Major changes*** |  |
| The rationale / justification of this article does not emphasize enough on the importance of providing data on the long-term outcomes of PPH: why is it important in terms of clinical practice and policy implications? o Lines 46-65: The introduction could be more developed. The rationale clearly explains that a knowledge gap exists and needs to be filled in this area of research, in view of the current knowledge on the matter (lines 51-61) but it fails to explain why it is important to identify what the long-term outcomes of PPH are, in terms of clinical practice but also in terms of policy implications. o Moreover, it is not clear whether the studies mentioned (ref 14-18) were investigating the longer-term outcomes of PPH or the immediate outcomes. | We have revised the introduction with these comments in mind. |
| Exploring women’s experience of PPH is key and including qualitative studies to this review is clearly a strength of the article. Yet, the experiences of care described in the eligibility criteria for the qualitative studies are more factors that could influence the occurrence of long-term consequences and ipso facto, do not answer directly to the main objective. The main objective of the review was to “synthesize the evidence about the long-term (…) consequences of PPH” but the paragraph on “Experiences of care” is not directly answering to this objective. It is an interesting part to understand the factors that could influence the occurrence of long-term consequences though but answers to a secondary objective. | Thank you for this important comment. We completely agree with the reviewer’s rationale and this is why we included care experiences in the review as care experiences of women and partners could influence the trajectory of long term consequences.  This was clearly set out as a research question in the PROSPERO protocol, but had been omitted from the manuscript in the interests of brevity. We have added to the introduction to justify the inclusion of the studies and to make this secondary objective explicit. |
| ***Minor changes/questions*** |  |
| Introduction  The writing of the three first sentences of the introduction (lines 46-51) lacks clarity in terms of linkages between ideas and the first sentences sometimes appear to have to no connection between one another. Concerning the writing, the linkage between ideas / sentences could be reinforced. Until reaching the paragraph on existing studies on the matter, it seems that the authors are presenting a succession of ideas without clear link between one another (definition – incidence – outcomes… but no written connection between these sentences). For example: Lines 48-49 about the incidence: how does it relate to the previous and following sentences? Not clear  The last sentence of the introduction is rather presenting the method to answer to the objective than presenting a clear research objective. Could be rephrased with more clarity. The last sentence of the introduction should clearly present the objective of this systematic review but here, instead, the authors are already presenting the methods used. | We have revised the introduction with these comments in mind, making a clearer link between sentences, improving the structure and adding more justification and rationale. The last two sentences of the introduction now clearly set out the objectives, without detailed reference to the methods used. |
| **Material and methods  Eligibility criteria:** - Table 1 is very helpful and very clear. Yet, the reader could benefit from two small clarifications: | Thank you for your comment about the eligibility criteria and Table 1. Our responses and changes made for each bullet point are as follows: |
| - The rationale for the definition of the exposure which is missing. How did the authors define the exposure definition of primary PPH? Could be interesting to explain further. | - We have added more detail in the text about the rationale behind the exposure definition. (l102-118 of unmarked manuscript) |
| - For exposure definition of primary PPH, did the author consider the way blood loss was estimated? Not clearly stated in the eligibility criteria. | - The method used to estimate blood loss was not part of the eligibility criteria and eligible papers were included regardless of how blood loss was estimated. We have added a sentence clarifying this. (l113 of unmarked manuscript) |
| - How did the author make sure that women had really suffered from PPH in qualitative studies without any eligibility criteria for exposure? | - A specific definition for PPH was not required for qualitative studies, as data about PPH are often collected directly from participants who take part in the study on the basis of a past experience of PPH. We only included papers in which it was explicitly stated that the participants were told by health professionals that they had PPH, or that specific interventions after PPH (e.g., emergency peripartum hysterectomy after PPH, arterial embolization after PPH) were performed. |
| - While reading the inclusion criteria for the outcomes, we need the information from the “time” cells to understand what “non-immediate” outcomes are. Merging the two lines of outcomes and time could help clarify this. | - Immediate outcomes are stated in the exclusion criteria so ‘non-immediate’ eligible outcomes are evident. We have retained “Time” as a separate criteria to follow the PICO(T) format/criteria of the PRISMA guideline. |
| - The systematic review focuses on longer-term outcomes of PPH with the main objective being to “synthesize the evidence about the long-term (…) consequences of PPH”. Yet, the inclusion criteria presented in table 1 consider that studies could be included with any follow-up duration after hospital discharge following PPH. Could we consider outcomes occurring at hospital discharge to be long-term outcomes (see table 4: study 33 – Drayton el al. 2016 and study 32 – Chessman et al 2018)? | - Please see our response above about the follow up period and our rationale for our definition of ‘longer-term’ outcomes. |
| **S1 File:**  What motivated this search strategy? | In thinking about the PECO/PICO(T), and in discussion with a health information specialist librarian, we considered it was appropriate to search only for our exposure of interest PPH and its synonyms. We did not include any population terms as the exposure of interest would only ever occur in pregnant women during childbirth. In order to optimise the sensitivity of the search and include papers where PPH might have been listed as one of a series of outcomes we also included potential interventions for PPH, blood transfusion and hysterectomy, alongside general morbidity terms. We also included pre-tested study filters for qualitative research and observational studies.  Our responses for each bullet point are as follows: |
| - Why not search all the synonyms of post-natal (OR postnatal OR peri-part OR peripart…) first, and then all the synonyms of hemorrhage and then link the two searches with AND? | Combining the search terms for postpartum AND for haemorrhage was too broad. We therefore applied the proximity search of postpartum adj2 (within 2 words of) haemorrhage. The decision to search line by line was a practical one as it allowed us to see the volume of references retrieved by each search line and assess what they contributed to the search. We could have search for (postnatal OR peri-part OR peripart...) adj2 (haemorrhage or hemorrhage)...) as one line but it would have made no difference to the references retrieved. |
| - Why search #20 till #38 and not apply filters? And why search these words only in title and abstract? | Lines 20-38 are acting as filters. The available filters for qualitative research are not optimised for sensitivity or specificity, as a result we applied our own (lines 20-23, S1 File) which uses a combination of textword (title/abstract), author keyword and subject headings. We based our observational filter on one designed by SIGN <https://www.sign.ac.uk/what-we-do/methodology/search-filters/> with slight adaptations to the needs of our review. |
| - Why not include the outcomes in the search strategy? | Outcomes were not included in the search terms as we did not have specific outcomes we were looking for and wanted to be more inclusive and minimise the chances of missing relevant outcomes of relevant papers. |
| **Results and discussion**  **Table 2**: Would be interesting to add the definition used for PPH in each study the follow-up time after PPH. | **Table 2.**  We have added the definition of PPH into Table 2. The information for follow-up time can be found in Table 4. |
| **Table 4:**   - Add a column with what has been studied could be helpful (PPH? EPH? Transfusion?) | **Table 4.**   - We have chosen not to include this extra column in Table 4 as the table already contains a lot of information and this would be duplicating information provided in Table 2. |
| - Error in the hemoglobin unit (study 32 – Chessman 2018) -> should be >9 g/dL or 7-9 g/dL or <7 g/dL and not >90 g/dL or 70-90 or <70 g/dL | - Thank you for spotting this error. We have corrected the haemoglobin units in Table 4. |
| - Revise column outcomes for psychological health outcomes (missing separation between some outcomes in the table). | - Thank you for spotting this. We have corrected some errors in table borders to separate the outcomes. |
| - For long term outcomes, no indication if patients had benefitted from therapy or not. Was it mentioned in some studies? It could really have an impact on the results. | - We did not indicate if the patients had benefitted from therapy or not as this is beyond the scope of this study. |
| - “It is important to provide women who had experienced PPH a thorough explanation and clear communication about their condition, treatments received including hysterectomy and its potential consequences.” (l371-373): Is it a result from the studies retrieved or a comment that should be included in the discussion part? Unclear in the way it is formulated. Should be stated from the perspective of women, from the eligible studies included in the analysis. | - Thank you for pointing out this ambiguity. This is the result from studies retrieved. We have rephrased this sentence to make it clearer. (l438-440 of unmarked manuscript) |
